# How employees respond to customer anger and sadness: emotional reciprocity and recovery strategies in service failures

**DOI:** 10.3389/fpsyg.2026.1795506

**Published:** 2026-05-20

**Authors:** Pushen Song, Yaqin Wang, Yang Yang, Ying Li

**Affiliations:** 1 Sichuan Conservatory of Music, Chengdu, China; 2 Guangzhou City Construction College, Guangzhou, China; 3 School of Mathematics, Southwestern University of Finance and Economics, Chengdu, China

**Keywords:** appraisal theory, customer anger, customer sadness, service employees, service recovery

## Abstract

Sadness and anger are among the most commonly experienced and expressed negative customer emotions in service failure encounters, yet their differential interpersonal effects on frontline employees remain underexplored. To address this gap, this study examines employees’ emotional and behavioral responses to customer sadness versus anger expressions, drawing on appraisal theory. Through two scenario-based experiments, we find that in the primary appraisal stage, employees experience other-concern emotions (such as empathy, sympathy, and compassion) in response to sad customers, and reciprocal anger in response to angry customers. In the secondary appraisal stage, employees strategically translate these emotions into recovery behaviors. Specifically, employees exert more spontaneous extra-role recovery effort for sad customers, whereas they provide comparable levels of mandatory in-role recovery for sad and angry customers. These findings contribute to the emotion literature on customer-employee interactions in service contexts, and offer valuable insights for service recovery management in the hospitality industry.

## 1 Introduction

Frontline employees in the hospitality industry regularly encounter customers expressing a range of negative emotions, including anger, sadness and disappointment, in response to service failures (Kranzbühler et al., 2020; Tronvoll, 2011). Service practitioners widely recognize that prompt and appropriate recovery are essential to mitigate customers’ negative emotions and prevent adverse consequences for firm performance (Smith and Bolton, 2002). Given their role as the primary point of contact in service interactions, how frontline employees interpret and respond to customer emotional cues is of both theoretical and practical importance (Cayla and Auriacombe, 2025; Chan and Wan, 2012; Glikson et al., 2019). This is particularly salient in the current era of artificial intelligence, where routine service tasks are increasingly automated, and the ability of human frontline employees to recognize and respond to nuanced customer emotional cues represents a distinctively human competency that warrants deeper investigation (Luo et al., 2025; Jose et al., 2026; Vorobeva et al., 2022).

Recent scholars have highlight the need for greater attention to customer emotional expressions within customer-employee interactions (Aeron and Rahman, 2025; Glikson et al., 2019). Existing research has largely concentrated on anger, the most prevalent negative emotion expressed by customers, documenting its adverse impacts on employees in the form of emotional work, reduced work effort (Cheng et al., 2020; Gabriel and Diefendorff, 2015; Rupp and Spencer, 2006), and even customer-directed sabotage (Wang et al., 2011). That said, a number of studies suggest that customer anger does not invariably produce negative employee responses. Glikson et al. (2019) and Jerger and Wirtz (2017) found that employees may prioritize service to angry customer who they perceived as having high status or power. Miron-Spektor et al. (2011) showed that exposure to customer anger can enhance their analytical but not creative task performance. Regarding the responses of employees in opposite way when they face angry customers, further understanding and exploration on this emotion and other influential factors are needed. By comparison, other negative emotions such as sadness, have received considerably less research attention (Van Kleef, 2016), despite evidence of their occurrence in hospitality contexts (Tuerlan et al., 2021). Sadness warrants closer examination precisely because, while it shares the same negative valence as anger, it carries distinct expressive cues and social meanings at the interpersonal level. Both emotions are commonly expressed outwardly during service failure encounters (Kranzbühler et al., 2020; Schwarzmüller et al., 2017), yet they differ markedly in their facial, physical, and verbal manifestations, enabling employees to distinguish between them (Van Kleef, 2016). As Anisman (2015) noted, humans as social animals are highly attuned to such interpersonal cues, and these subtle differences in expression may trigger meaningfully different emotional, cognitive and behavioral responses. This raises the central question guiding the present research: do customer sadness and anger expressions constitute distinct interpersonal cues that elicit different responses from frontline employees?

Drawing on appraisal theory, this research takes a comparative approach to examine how customer sadness versus anger expressions shape employees’ emotional and behavioral responses. We propose that through a two-stage appraisal process, employees first evaluate the meaning of customer expressions in terms of customers’ emotional state and social intention, and then assess the behavioral options available to them (Lazarus and Folkman, 1984). In the primary appraisal stage, employees interpret angry customers are expressing blame and potential confrontation, whereas sad customers are perceived as experiencing personal loss and seeking support without accusation (Sinaceur et al., 2015; Van Kleef et al., 2008). These appraisals give rise to distinct emotional reactions of employees: anger toward angry customers, and other-concern emotions toward sad customers. In the secondary appraisal stage, employees seek behavioral outlets for these reactions, but they must do so within the constraints of their service role, which defines the range of coping strategies available to them (Jerger and Wirtz, 2017; Schepers and van der Borgh, 2020). Accordingly, we focus employees’ behavioral responses on two forms of service recovery, that is in-role and extra-role recovery.

This research makes four contributions. First, it offers a comparative analysis of customer sadness and anger as distinct interpersonal cues, examining their differential effects on frontline employees. While the existing literature has focused predominantly on anger (Van Kleef, 2016), this study broadens the scope of emotion research in service contexts by attending to both emotions and their divergent interpersonal consequences. Second, the study demonstrates that employees draw strategically on in-role and extra-role recovery behaviors as differentiated coping responses to customer emotional expressions. This contributes to service recovery research by moving beyond normative accounts of what employees “should do” to examine what employees “would do” in emotionally charged interactions (Schepers and van der Borgh, 2020; Van Vaerenbergh et al., 2019). In doing so, the research also helps reconcile the mixed findings in the customer anger literature. Third, by applying appraisal theory to the interpersonal dynamics of service failure, this research complements prior work that has focused on customers’ own appraisal and coping processes (e.g., Gelbrich, 2010; González-Gómez et al., 2021), and extends the theory’s application to the employee side of the service encounter. Fourth, by drawing the pathway from customer sadness through employees’ other-concern emotions to extra-role recovery behaviors, the study sheds light on the origins and dynamics of employee altruism in episodic service interactions (Dutton et al., 2014; Wieseke et al., 2012).

In the remainder of this paper, we first review research on customer sadness and anger, examining their distinctions in expressive cues and interpersonal meaning. We then develop hypotheses regarding the differential effects of these two emotions on frontline employees’ reactive emotions and service recovery behaviors, drawing on appraisal theory. The hypotheses are tested through two scenario-based experiments. In the discussion section, we discuss the theoretical and practical implications of the research, as well as the study’s limitations and directions for future research.

## 2 Literature background and hypotheses development

### 2.1 Customer anger and sadness

Negative emotions arise when people perceive their goals to be blocked or unattainable (Frijda et al., 1989). Both anger and sadness are common emotional responses to service failure, as customers experience hindrances to their consumption goals and unmet expectations (Kranzbühler et al., 2020; Tronvoll, 2011). Despite sharing the same negative valence, the two emotions can be distinguished along the appraisal dimension of blame attribution (Roseman et al., 1994). Blame attribution refers to the extent to which hold service providers accountable for a negative outcome (Gelbrich, 2010). Anger tends to arise when customers attribute the failure to the firm or its employees, perceiving them as responsible for a preventable event (Albrecht et al., 2017; Gelbrich, 2010). Sadness, by contrast, tends to emerge when customers do not assign blame to the provider but instead attribute the failure to circumstances beyond the firm’s control, such as bad weather or the behavior of other customers (Becker et al., 2018; Sinaceur et al., 2015). Thus, the same service failure event can elicit either anger or sadness depending on how customers apportion blame (Gelbrich, 2010; Sinaceur et al., 2015).

The prevalence of customer anger in service failure is well documented (Cheng et al., 2020; Surachartkumtonkun et al., 2015). Customer sadness, while less common, has also been observed across a range of service settings. Research in the hotel context has shown that dissatisfied customers may feel sad or resigned, particularly when they attribute failures to factors outside the firm’s control (Tuerlan et al., 2021; Gelbrich, 2010). Similarly, airline passengers have been found to experience sadness in response to luggage loss or food dissatisfaction (Scherer and Moors, 2019; Xu et al., 2019). Using the critical incident technique, Tronvoll (2011) found that sadness accounted for 30.5% of emotions reported in service contexts, underscoring its relevance as a distinct emotional response.

Beyond the internal experience, customers actively communicate their emotions through expressive cues during social interactions (Van Kleef, 2016). Among negative emotions, anger and sadness are particularly likely to be expressed interpersonally, as both call for social attention and response (Kranzbühler et al., 2020; Schwarzmüller et al., 2017). The two emotions are nonetheless distinguishable through distinct expressive channels. Physically, angry customers tend to display confrontational postures and facial expressions such as a glare, a scowl, tightly closed lips, and flared nostrils (Jerger and Wirtz, 2017; McColl-Kennedy et al., 2009), and verbally they are characterized by raised tone and scolding remarks (Sinaceur et al., 2015). Sad customers, in contrast, display a somber expression with lowered eyebrows and an upturned cheek, and tend to soften their voice or sigh (Sinaceur et al., 2015). Given the richness of emotional exchange in service encounters (Groth et al., 2019), these expressive cues are readily perceived by frontline employees and carry meaningful social information that shapes their subsequent responses (Lelieveld et al., 2012; Liu et al., 2019).

### 2.2 Employee responses to customer anger versus sadness

Considerable scholarly attention has been devoted to the effects of customer anger on frontline employees, with evidence indicating that such expressions are detrimental to employee well-being and performance. Employees confronted with angry customers are readily irritated, which can escalate into an “anger spiral” within the dyadic service interaction (Dudenhöffer and Dormann, 2013; Gabriel and Diefendorff, 2015). Consequently, employees may reduce their service effort (Cheng et al., 2020; Goldberg and Grandey, 2007), engage in more surface acting (Yoo et al., 2025; Gabriel and Diefendorff, 2015; Rupp and Spencer, 2006), and in some cases direct sabotage toward the offending customer (Nyjer et al., 2026; Wang et al., 2011). However, there are a smaller body of work suggesting that customer anger is not invariably counterproductive. For instance, Glikson et al. (2019) and Jerger and Wirtz (2017) found that employees may increase restitution efforts when an angry customer is perceived as having high status or power, while Miron-Spektor et al. (2011) showed that exposure to customer anger can improve routine task performance, even as it undermines creative performance. These mixed findings underscore the need for further research into the interpersonal effects of customer anger on employees.

By comparison, customer sadness has received far less scholarly attention despite being a comparably prevalent emotional response to service failure. As noted earlier, sadness and anger share the same negative valence but differ markedly in their intrapersonal appraisal patterns and interpersonal expressive cues (Kranzbühler et al., 2020; Roseman et al., 1994). This contrast makes a comparative examination of their interpersonal effects both theoretically meaningful and practically informative. The following section develops this comparison in depth, tracing how customer expressions of sadness and anger shape employee responses through distinct pathways.

### 2.3 Hypothesis development

#### 2.3.1 Appraisal theory

Appraisal theory posits that individuals’ emotional and behavioral responses are determined by a two-stage evaluations of the target event (Lazarus and Folkman, 1984). In the primary appraisal stage, individuals assess the nature of the event and its relevance to their goals, which therefore triggers an immediate affective reaction (Lazarus, 1991). In the subsequent secondary stage, individuals engage in deeper cognitive processing, drawing on available resources to evaluate their capacity to cope with the event (Lazarus, 1991; Lazarus and Folkman, 1984). Depending on situational constraints, individuals may act on their initial emotional reactions from the primary stage or strategically regulate their behavioral responses following secondary appraisal.

**Figure 1:**
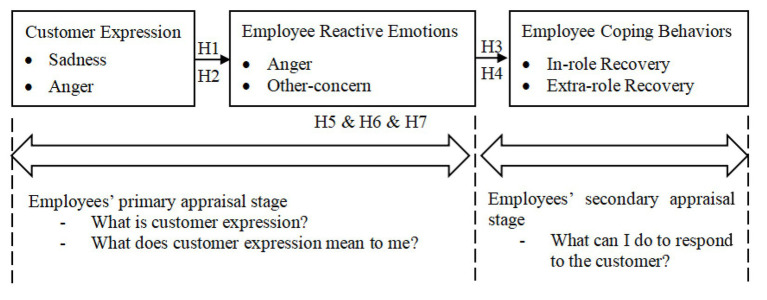
The conceptual model of this research.

For frontline employees, customer expressions of negative emotion constitute a goal-incongruent event, as they violate the expectation of harmonious service interactions and trigger immediate emotional reactions in the primary appraisal stage (Luo et al., 2021). At the same time, as organizational representatives, employees must weigh their role responsibilities and the potential consequences of allowing emotion-driven impulses to guide their recovery actions (Fu et al., 2022). Appraisal theory thus provides a valuable framework for understanding how customer emotional expressions shape employees’ service recovery responses (see Figure 1). Specifically, the immediate affective reactions generated in the primary appraisal stage, in combination with employees’ assessment of their coping potential, jointly inform the recovery approach adopted in the secondary stage. In the primary appraisal stage, employees differentiate between customer anger and sadness based on their distinct expressive cues, giving rise to qualitatively different emotional reactions (i.e., reciprocal anger versus other-concern emotions) as proposed in H1 and H2. In the secondary appraisal stage, these emotional reactions guide employees’ selection of coping behaviors, yet are constrained by role expectations, ultimately resulting in differentiated in-role versus extra-role recovery, as captured in H3 through H7.

#### 2.3.2 Effect of customer expression on employees’ reactive emotions

During service failures, employees can quickly register both the verbal content and physical expressions of customers, enabling them to grasp the emotional state of the person before them. Research shows that basic emotions such as anger and sadness are reliably recognized across cultures (Van Kleef, 2016; Tottenham et al., 2009; Arici et al., 2022). Through a process of “reverse appraisal,” employees distill meaningful information from these emotional displays, inferring the underlying causes and intentions behind customer expressions (Hareli et al., 2009). For instance, employees observing customer anger are likely to infer blame directed at the firm or its staff, whereas those observing sadness tend to perceive a customer in a helpless state, absent of accusation (Sinaceur et al., 2015). Employees also anticipate what these emotional signals imply for the customer’s subsequent behavior toward them (Van Kleef, 2016). Anger is typically read as a confrontational signal with potential for conflict (Hareli et al., 2009; Lelieveld et al., 2012; Sinaceur et al., 2015), while sadness is generally interpreted as a support-seeking signal, indicating distress and a need for assistance rather than confrontation (Becker et al., 2018; Yagil and Medler-liraz, 2013).

According to emotional reciprocity theory, people tend to respond in kind to perceived emotions, though not necessarily by mirroring them directly. Emotional responses often follow a functional adaptation process, shaped by the perceived social function of the initiating emotion (Sinaceur et al., 2015). When facing an angry customer, employees perceive the expression as an attack or demand, triggering defensive reciprocity and a corresponding experience of reciprocal anger (Glikson et al., 2019; Rupp and Spencer, 2006). Customer sadness, by contrast, functions as a support-seeking signal that activates social bonding processes, eliciting functional reciprocity rather than direct emotional mirroring (Becker et al., 2018; Yagil and Medler-liraz, 2013). Accordingly, employees responding to a sad customer are unlikely to experience sadness themselves; instead, they are more likely to experience other-concern emotions, namely sympathy, empathy, and compassion.

Sympathy, empathy, and compassion, while conceptually distinct, collectively constitute what scholars have termed other-concern or other-directed emotions. Empathy involves the cognitive and affective process of understanding and sharing another’s emotional state; sympathy reflects an affective concern for another’s well-being without necessarily sharing their feelings (Wieseke et al., 2012); and compassion extends this concern toward a motivation to actively alleviate the other’s suffering (Dutton et al., 2014). Despite these distinctions, all three emotions share a common functional orientation, directing attention and prosocial resources toward another person’s welfare in response to perceived vulnerability or distress (Van Kleef et al., 2008; Sinaceur et al., 2015). In service failure encounters, this shared orientation suggests that other-concern emotions operate through a common functional pathway, facilitating social support and cooperative responses toward customers in need (Allred et al., 1997; Sinaceur et al., 2015). Therefore, we propose:

*H1*: Employees are more likely to experience anger when interacting with an angry customer than with a sad customer.

*H2*: Employees are more likely to experience other-concern emotions when interacting with a sad customer than with an angry customer.

It is worth noting that employees’ anger and other-concern responses are not simply opposite ends of a single emotional continuum, but rather reflect qualitatively distinct reciprocity mechanisms. Specifically, anger represents a case of defensive reciprocity, referring to a self-protective response triggered by the perceived threat and blame embedded in the customer’s aggressive expression. In contrast, other-concern emotions reflect functional reciprocity, a socially adaptive response that mirrors the support-seeking nature of the customer’s sadness without directly replicating the emotion itself (Sinaceur et al., 2015; Becker et al., 2018). These two mechanisms differ not only in their affective content but also in their motivational orientation: defensive reciprocity orients employees toward self-protection, whereas functional reciprocity orients them toward prosocial action directed at the other. This distinction is consequential for the secondary appraisal stage, where employees must translate their immediate emotional reactions into concrete behavioral responses within the constraints of their service role, a process we examine in the following section.

#### 2.3.3 Effect of customer expression on employees coping behaviors

Given that employees’ primary emotional reactions vary with the type of customer expression they encounter, a natural question arises: do these emotional reactions translate directly into behavior, following a feeling-for-doing logic (Zeelenberg et al., 2008)? That is, do employees who feel reciprocal anger toward angry customers reduce their recovery effort, while those who feel other-concern toward sad customers invest more (Cheng et al., 2020; Rafaeli et al., 2012; Sinaceur et al., 2015; Van Kleef et al., 2008)? We acknowledge the influence of primary appraisal emotions on subsequent behavior, but argue that what employees want to do does not always determine what they can do.

According to the appraisal theory, employees engage in deeper cognitive assessment to determine how best to respond to emotional customers given their available resources and role constraints in the secondary appraisal stage (Fu et al., 2022; Sahaf and Fazili, 2024). To understand the range of behavioral options available, it is useful to distinguish between two types of service recovery. In-role recovery behaviors refer to the core, mandatory responsibilities of frontline employees, primarily involving problem-solving and courteous treatment of the customer (van der Heijden et al., 2013). Because these behaviors are required, failure to perform them can result in punishment or termination if the situation escalates (Bettencourt and Brown, 1997). Extra-role recovery behaviors, by contrast, are discretionary and go beyond formal job requirements to offer personalized assistance or emotional care (Bettencourt and Brown, 1997; Chan and Wan, 2012; Schepers and van der Borgh, 2020). There is no penalty for omitting them and no guaranteed reward for providing them.

These two behavioral categories define the coping space available to employees. When facing angry customers, employees may feel irritated and less inclined to help, yet they must suppress these reactions to fulfill mandatory in-role recovery obligations, as failing to do so jeopardizes their professional standing (Jerger and Wirtz, 2017). At the same time, the discretionary nature of extra-role recovery allows employees to withhold this additional effort without repercussion (Wang et al., 2012). Conversely, customer sadness activates other-concern emotions that not only sustain routine in-role recovery but also motivate employees to extend themselves through spontaneous extra-role helping (Becker et al., 2018; Sinaceur et al., 2015). Therefore, we hypothesize:

*H3*: Customer anger expression versus sadness expression leads to similar level of in-role recovery behaviors by employees.

*H4*: Customer sadness expression promotes more extra-role recovery behaviors by employees than anger expression.

While employees’ behavioral responses are constrained by role demands, their reactive emotions nonetheless play an important mediating role in this process. As Lazarus (1991) argued, even when behavioral responses do not directly mirror initial emotional reactions, emotions remain a fundamental element throughout the appraisal and coping process. Following the feeling-for-doing logic (Zeelenberg et al., 2008; Frijda, 1986), both anger and other-concern emotions are expected to shape the intensity and form of employees’ recovery efforts, even within the boundaries set by role obligations. Therefore, we hypothesize:

*H5*: Employees’ anger and other-concern emotions mediate the relationship between customer emotional expressions (sadness vs. anger) and in-role recovery behaviors.

*H6*: Employees’ anger and other-concern emotions mediate the relationship between customer emotional expressions (sadness vs. anger) and extra-role recovery behaviors.

Given that other-concern emotions foster prosocial behavior and cooperative responding (Allred et al., 1997; Wieseke et al., 2012), and given the inherently discretionary and altruistic nature of extra-role recovery, we further anticipate that the indirect effect of customer emotional expressions through employees’ other-concern emotions will be stronger for extra-role than for in-role recovery.

*H7*: The indirect effect of customer emotional expression through employees’ other-concern emotions is stronger on extra-role recovery behaviors than on in-role recovery behaviors.

## 3 Research method

To test our hypotheses, we followed Dixon et al. (2017), and conducted two scenario-based experiments for several reasons. First, descriptive vignettes allow us to construct controlled situations that closely map onto our theoretical framework, which is particularly suitable for studying the nuanced emotional dynamics of service interactions. Second, fictional scenarios minimize memory bias and self-reporting bias among participants. Third, the controlled setting enhances internal validity by ensuring all participants engage with a common experience. Fourth, ecological validity was addressed through a series of pretests conducted prior to the formal studies to verify the effectiveness of the scenarios and manipulations.

Study 1 aimed to test employees’ emotional responses in the primary appraisal stage when confronted with different customers expressions. The failure scenario was adapted from Gelbrich (2010), and business students served as participants. Study 2 extended this work by recruiting actual frontline hotel employees via Prolific, using a newly developed service failure scenario in a hospitality context. This study replicated the emotional response findings from Study 1, further examined employees’ behavioral coping responses in the secondary appraisal stage, tested the mediating effects of reactive emotions, and eliminated the potential confounding influence of perceived failure severity.

### 3.1 Study 1

#### 3.1.1 Study design

Study 1 employed a single-factor between-subjects design with three conditions (customer emotional expression: anger vs. sadness vs. neutrality) to examine the impact of customer emotional expressions on employees’ reactive emotions. The neutrality condition served as a baseline for comparison. The failure scenario was adapted from Gelbrich (2010), in which a customer complains to a frontline employee (see Appendix A for detailed scenario). As Gelbrich (2010) confirmed, this scenario can plausibly elicit either anger or sadness depending on customers’ attributions. We made targeted adaptations to highlight the customer’s emotional expression while preserving the original scenario structure. Customer emotions were manipulated using facial photographs, consistent with evidence that hotel employees can reliably recognize emotions from facial cues (Koc and Boz, 2020).

The facial stimuli were drawn from the NimStim Face Stimulus Set (Tottenham et al., 2009), a widely used standardized database comprising 646 photographs depicting six basic emotions as well as calmness and emotional neutrality. Prior to the formal study, we conducted two pretests to assess the effectiveness of the scenario and the three facial photographs used as stimuli.

#### 3.1.2 Pretest

For the scenario pretest, 54 undergraduate students (62% female) were presented with the text-based scenario and asked to rate the extent to which they would feel anger and sadness in that situation. Results indicated comparable levels of anger and sadness [*M*_anger_ = 4.50, *M*_sadness_ = 4.17, *t*(53) = 1.41, *p* = 0.164], confirming that the scenario could plausibly elicit either emotion depending on individual attribution, and thus providing a suitable basis for the subsequent manipulation.

In the pretest of facial pictures, 90 participants were recruited via MTurk, an online platform increasingly used in behavioral research for its efficiency and scalability (Mason and Suri, 2012). Participants viewed three photographs displaying anger, sadness and neutrality expressed by the same individual (see Appendix B for the manipulation), and rated each using the measures from Cheshin et al. (2018). Results confirmed the validity of all three stimuli. The sadness picture was rated as conveying significantly more sadness than both the anger picture [*M*_sadness_ = 5.27, SD = 1.42; *M*_anger_ = 3.10, SD = 1.49; *t*(58) = 5.77, *p* < 0.001], and the neutrality picture [*M*_neutrality_ = 3.67, SD = 1.23; *t*(58) = 4.66, *p* < 0.001]. The anger picture was rated as conveying significantly more anger than both the sadness picture [*M*_anger_ = 5.50, SD = 1.23; *M*_sadness_ = 3.10, SD = 1.24; *t*(58) = 7.54, *p* < 0.001] and neutrality picture [*M*_neutrality_ = 3.70, SD = 1.05, *t*(58) = 6.10, *p* < 0.001]. There was no significant difference in the perceived emotional intensity for the sadness and anger pictures [*M*_anger_ = 5.27, SD = 1.28; *M*_sadness_ = 5.23, SD = 1.09; *t*(58) = 0.13, *p* = 0.897], but both were rated as significantly more intense than the neutrality picture [*M*_anger_ = 5.27, SD = 1.28; *M*_neutrality_ = 3.87, SD = 1.04, *t*(58) = 4.64, *p* < 0.001; *M*_sadness_ = 5.23, SD = 1.09; *M*_neutrality_ = 3.87, SD = 1.04, *t*(58) = 4.93, *p* < 0.001].

#### 3.1.3 Participants and procedure

A total of 144 European business students participated Study 1 for credits (28% female). All participants were asked to imagine themselves as a hotel receptionist and to read a scenario which a customer approached them to complain about a noise disturbance during their stay. They were then randomly assigned to one of three conditions, in which the customer displayed either sadness, anger, or a neutral expression. Following exposure to the scenario and facial stimulus, participants completed measures of their reactive anger and other-concern emotions toward the customer. The study concluded with manipulation checks of the facial stimuli.

#### 3.1.4 Measures

All measures used 7-pont Likert scale and were from established literature (see Appendix C). Employees’ other-concern emotions were assessed through a composite measure of sympathy, empathy, and compassion, adapted from Van Kleef et al. (2008) and Sinaceur et al. (2015). Four items measured employees’ anger from Du et al. (2014). Both constructs demonstrated acceptable reliability with Cronbach’s *α* above 0.89.

#### 3.1.5 Results

##### 3.1.5.1 Manipulation checks

The results confirmed that the manipulations were successful. Participants in the sadness condition rated the customer significantly sadder than those in both the anger condition [*M*_sadness_ = 5.33, SD = 1.40; *M*_anger_ = 2.92, SD = 1.33; *t*(94) = 8.64, *p* < 0.001] and the neutrality condition [*M*_neutrality_ = 3.52, SD = 1.34; *t*(95) = 6.48, *p* < 0.001]. Conversely, participants in the anger condition rated the customer more angry than those in both the sadness condition [*M*_anger_ = 5.35, SD = 1.16; *M*_sadness_ = 3.06, SD = 1.42; *t*(95) = 8.67, *p* < 0.001] and the neutrality condition [*M*_neutrality_ = 3.77, SD = 1.19; *t*(95) = 6.61, *p* < 0.001]. Moreover, participants in the sadness condition and anger condition perceived the similar level of emotional intensity of our manipulation [*M*_sadness_ = 5.36, SD = 0.98; *M*_anger_ = 5.13, SD = 1.12; *t*(95) = 1.16, *p* = 0.25], and both were rated as significantly more intense than the neutrality condition [*M*_sadness_ = 5.36 vs. *M*_neutrality_ = 3.88, *t*(95) = 6.90, *p* < 0.001; *M*_anger_ = 5.13 vs. *M*_neutrality_ = 3.88; *t*(95) = 5.51, *p* < 0.001].

##### 3.1.5.2 Main effects

**Figure 2:**
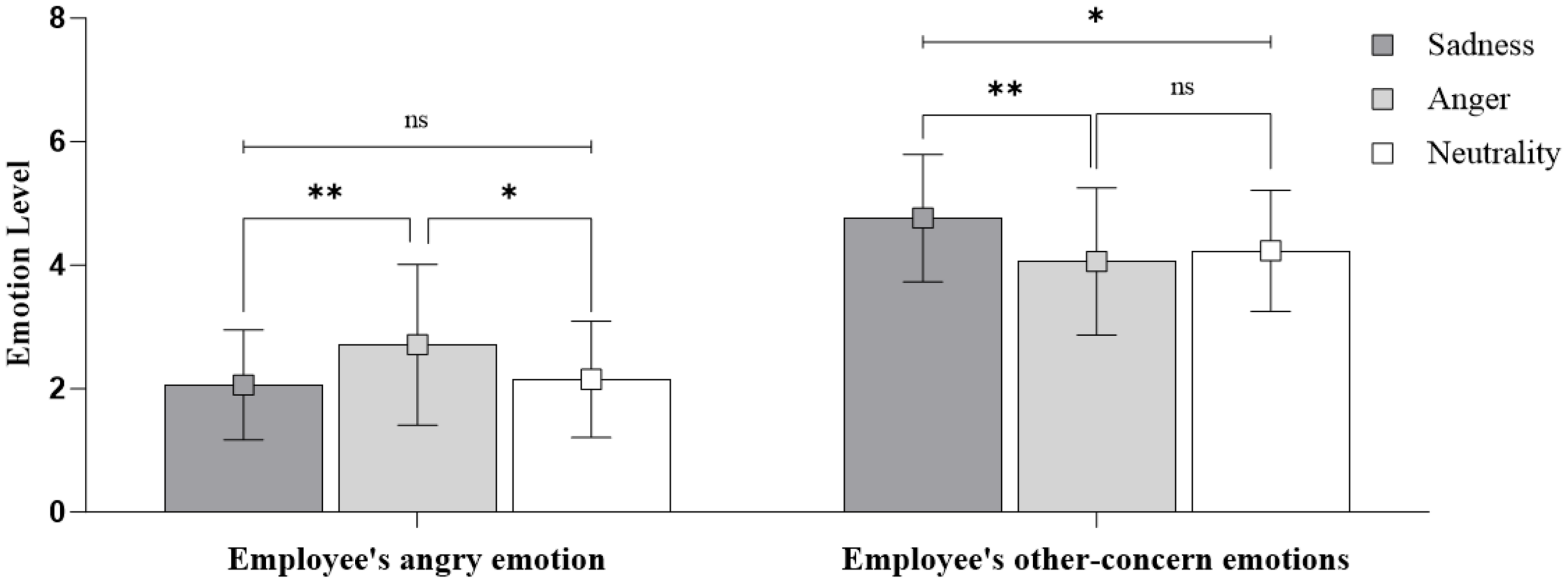
The effects of customer expressions on employees’ reactive emotions in Study 1. ns represents non-significant difference at *p* > 0.10, * represents significant difference at *p* < 0.05, ** represents significant difference at *p* < 0.01, *** represents significant difference at *p* < 0.001.

ANOVA analyses were conducted with customer emotional expressions as independent variable, and employees’ reactive emotions towards customer as dependent variables. Results revealed the main effect of customer emotional expressions on employees’ angry towards the customer [*F*(2, 143) = 5.40, *p* < 0.01]. As shown in Figure 2, employees reported greater reciprocal anger toward the angry customer than toward the sad customer [*M*_anger_ = 2.71, SD = 1.30; *M*_sadness_ = 2.06, SD = 0.89; *p* < 0.01, *η*^2^ = 0.070] and neutral customer (*M*_neutrality_ = 2.15, SD = 0.94; *p* < 0.05). Thus, H1 was supported.

A significant main effect was also found for employees’ other-concern emotions [*F*(2, 143) = 5.75, *p* < 0.01]. As Figure 2 shown, Employees reported greater other-concern toward the sad customer than toward both the angry customer (*M*_sadness_ = 4.76, SD = 1.03; *M*_anger_ = 4.06, SD = 1.19; *p* < 0.01, *η*^2^ = 0.074), and the neutral customer (*M*_neutrality_ = 4.23, SD = 0.98; *p* < 0.05). Thus, H2 was supported.

#### 3.1.6 Discussion

Study 1 provides initial evidence that customer emotional expressions shape employees’ reactive emotions in the primary appraisal stage, in support of H1 and H2. Customer sadness elicited greater other-concern emotions, while customer anger elicited greater reciprocal anger. An unexpected but noteworthy finding, however, is that employees consistently reported higher levels of other-concern than anger across all conditions, including the neutral condition (*M*_neutrality_ = 4.23 vs. *M*_neutrality_ = 2.15, *p* < 0.05). This may reflect internalized role expectations in service contexts, whereby employees maintain a compassionate orientation toward customers regardless of the emotional expression displayed. Additionally, the use of business students as proxies for frontline employees introduces limitations on ecological validity, as students may lack the professional socialization and accumulated service experience that shape how actual employees interpret and respond to customer emotional cues. To address these concerns, Study 2 recruited actual frontline hotel employees and employed a newly developed scenario to replicate and extend the findings.

### 3.2 Study 2

#### 3.2.1 Study design

Study 2 was conducted to replicate and extend the emotional response findings from Study 1 using actual hotel employees, and to test the complete hypothesized model including the mediating roles of reactive emotions and employees’ behavioral coping responses. Study 2 additionally tried to rule out possible confounding variable of perceived severity of failure. Prior research has shown that observers may reframe events through others’ emotional expressions and adjust their responses accordingly (van Doorn et al., 2012; Hillebrandt and Barclay, 2017), suggesting that participants may perceive different levels of failure severity depending on whether the customer appears angry or sad. Given that perceived severity is known to influence employees’ recovery responses (Fu et al., 2022), it was included as a control variable.

To select an appropriate scenario, we developed six service failure scenarios spanning hotel, restaurant, and airline contexts and conducted a within-subjects pretest with 36 MTurk participants, who rated the extent to which each scenario would make them feel sad or angry as a customer. A scenario depicting limited access to pool facilities at a hotel was selected (see Appendix A for detailed scenario), on the basis that it elicited comparable levels of sadness and anger [*M*_sadness_ = 4.00, SD = 1.45; *M*_anger_ = 4.28, SD = 1.54; *t*(35) = 0.93, *p* = 0.359], with no significant correlation between the two emotions (*γ* = 0.28, *p* = 0.098).

The facial photographs used to represent customer expressions were identical to those in Study 1. To enhance realism, a verbal statement was added alongside each image. In the sadness condition, “I am really sad because the pool in the hotel was crowded…”; in the anger condition, “I am really angry because the pool in the hotel was crowded!”.

#### 3.2.2 Participants and procedure

Study 2 employed a single-factor between-subject design with two conditions (customer expression: anger vs. sadness). A total of 160 frontline hotel employees (57.5% female) were recruited via Prolific, an online research platform known for providing access to diverse and high-quality participant samples (Peer, 2024). Participants were randomly assigned to either the anger or sadness condition. The procedure followed the same structure as Study 1.

#### 3.2.3 Measures

All measures used 7-point Likert scales. The measure of in-role recovery were adapted from van der Heijden et al. (2013), encompassing problem-solving and courtesy showing. Extra-role recovery was measured by four items adapted from Chan and Wan (2012). Measures of employees’ reactive emotions (i.e., anger emotion and other-concern emotions) were consistent with Study 1. Perceived failure severity was assessed using the scale from Grégoire et al. (2009). All scales exhibited acceptable reliability, with Cronbach’s αs ranging from 0.82 to 0.95 (see Appendix C).

#### 3.2.4 Results

##### 3.2.4.1 Manipulation checks

Participants in the sadness condition evaluated the customer sadder than participants in the anger condition [*M*_sadness_ = 5.31, SD = 1.18; *M*_anger_ = 2.56, SD = 1.56; *t*(158) = 12.60, *p* < 0.001]. Conversely, participants in the anger condition evaluated the customer angrier than those in the sadness condition [*M*_anger_ = 5.85, SD = 1.04; *M*_sadness_ = 2.59, SD = 1.48; *t*(158) = 16.10, *p* < 0.001].

##### 3.2.4.2 Main effects

**Figure 3:**
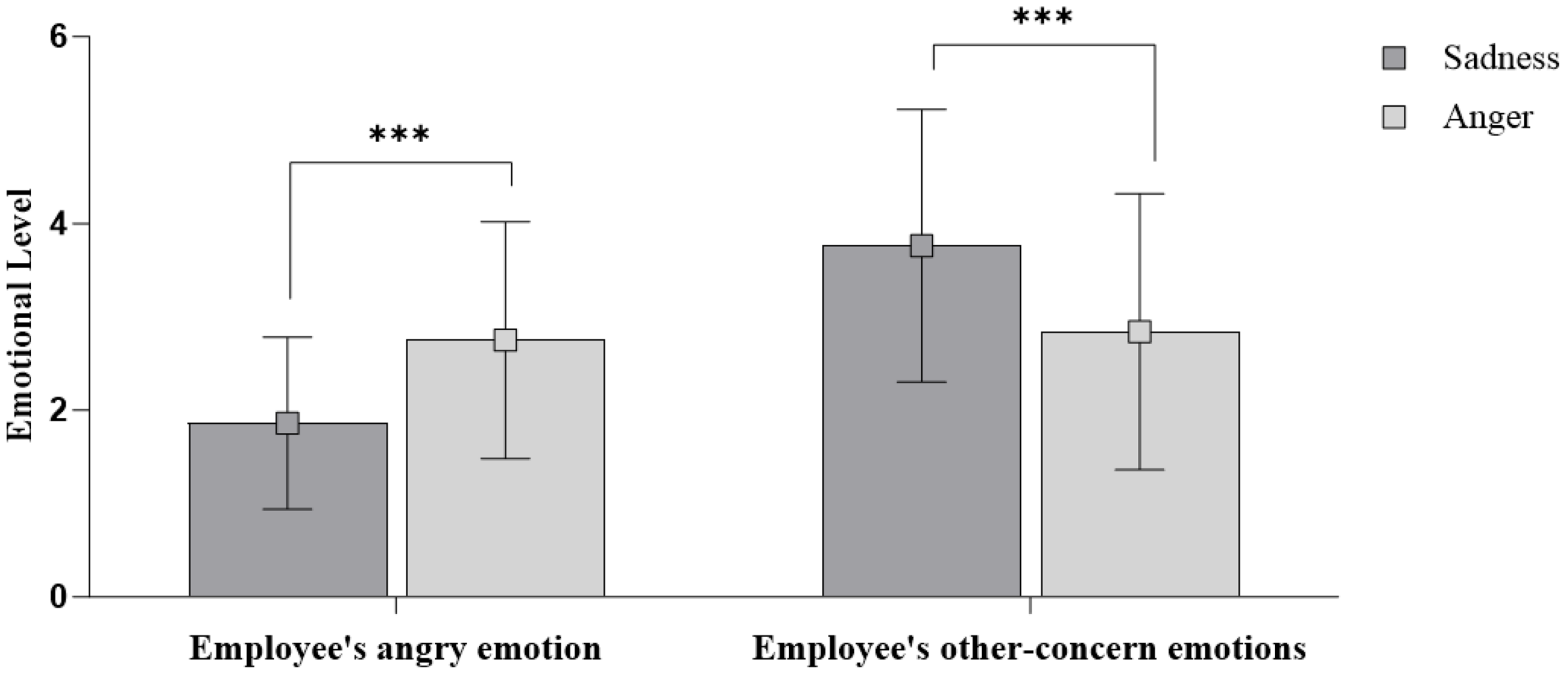
The effects of customer expressions on employees’ reactive emotions in Study 2. ns represents non-significant difference at *p* > 0.10, * represents significant difference at *p* < 0.05, ** represents significant difference at *p* < 0.01, *** represents significant difference at *p* < 0.001.

We conduct an ANCOVA analysis by including gender and perceived failure severity as the control variables. As shown in Figure 3, the effect of customer expressions on employees’ anger was significant [*F*(1,158) = 27.53, *p* < 0.001]. While neither the gender [*F*(1,158) = 3.30, *p* = 0.071] nor perceived severity [*F*(1,158) = 0.57, *p* = 0.451] were significant. Employees reported greater anger in response to the angry customer than the sad customer (*M*_anger_ = 2.75, SD = 1.27; *M*_sadness_ = 1.86, SD = 0.92; *p* < 0.001, *η*^2^ = 0.148). Therefore, H1 was supported again. Customer expression also had a significant effect on employees’ other-concern emotions [*F*(1, 158) = 27.54, *p* < 0.001], with the sad customer eliciting greater other-concern than the angry customer [*M*_sadness_ = 3.76, SD = 1.46; *M*_anger_ = 2.84, SD = 1.48; *p* < 0.001, *η*^2^ = 148], in support of H2. Gender did not significantly affect other-concern [*F*(1, 158) = 0.90, *p* = 0.344], though perceived failure severity did [*F*(1, 158) = 79.15, *p* < 0.001].

**Figure 4:**
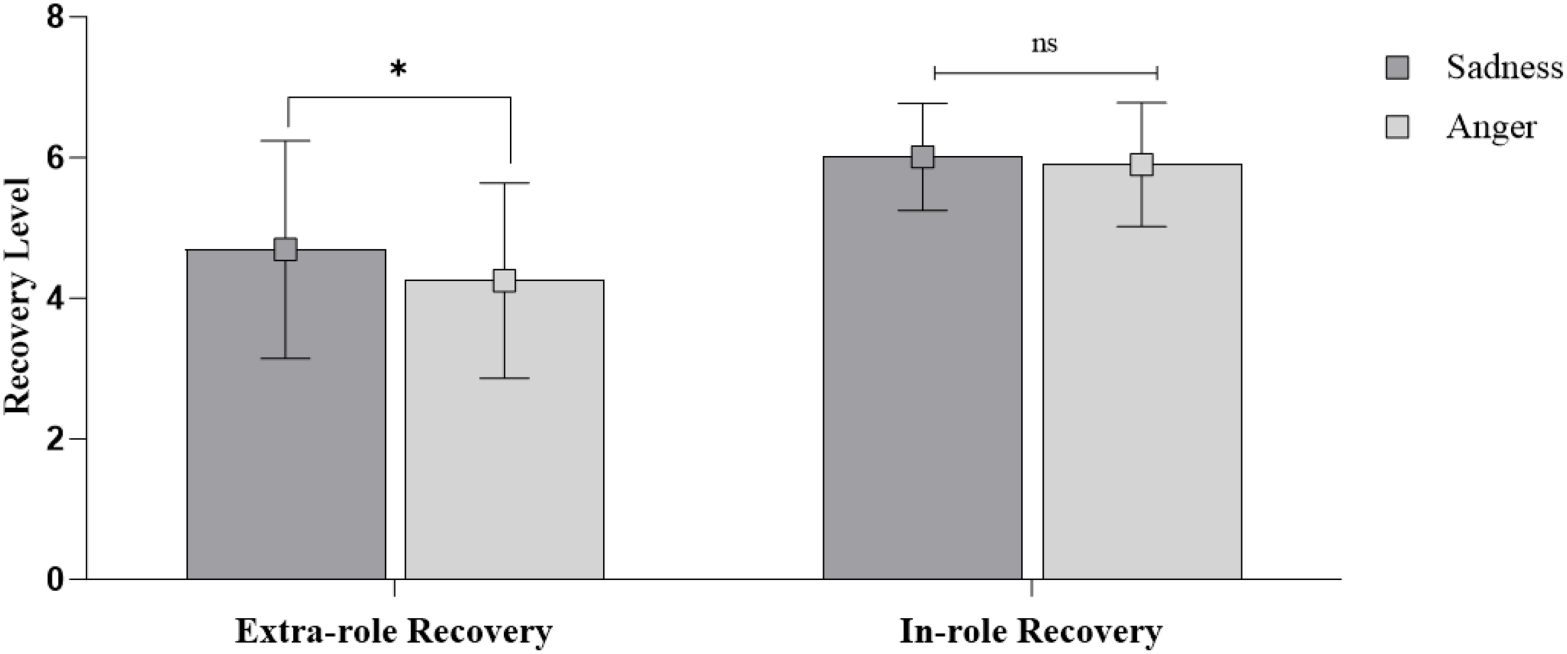
The effects of customer expressions on employees’ recovery behaviors in Study 2. ns represents non-significant difference at *p* > 0.10, * represents significant difference at *p* < 0.05, ** represents significant difference at *p* < 0.01, *** represents significant difference at *p* < 0.001.

For in-role recovery, the ANCOVA revealed no significant difference between two conditions [*M*_sadness_ = 6.01, SD = 0.76; *M*_anger_ = 5.90, SD = 0.88; *F*(1, 158) = 0.57, *p* = 0.451] (see Figure 4). Furthermore, neither control variables were significant. Therefore, H3 was supported. For extra-role recovery, a significant effect of customer expression was found after controlling for gender and severity [*F*(1, 158) = 4.94, *p* < 0.05]. Employees facing a sad customer reported greater willingness to exceed role requirements than those facing an angry customer [*M*_sadness_ = 4.69, SD = 1.15; *M*_anger_ = 4.25, SD = 1.39; *p* < 0.05, *η*^2^ = 0.030; see Figure 4], supporting H4. Gender was not significant [*F*(1, 158) = 2.33, *p* = 0.129], while perceived failure severity was significant [*F*(1, 158) = 11.61, *p* < 0.01].

##### 3.2.4.3 Mediation effects

To test the mediating effects of employees’ reactive emotions, we conducted the PROCESS mediation test with Model 4 with gender and perceived severity included as covariates. In addition to rule out the alternative explanation that employees may infer different levels of failure severity from customer anger and sadness, we also included perceived severity in the mediating test. As Table 1 showed, we confirmed the mediating effects of employees’ anger (*b* = 0.20, 95% CI [0.10, 0.33]) and other-concern emotions (*b* = 0.12, 95% CI [0.04, 0.22]) between customer expressions and in-role recovery. Similarly, the mediating effects of employees’ anger (*b* = 0.19, 95% CI [0.05, 0.39]) and other-concern emotions (*b* = 0.44, 95% CI [0.21, 0.73]) between customer expressions and extra-role recovery were also significant. When controlling all mediating influences, the direct effect of customer expressions on extra-role recovery was not significant (*b* = 0.40, 95% CI [−0.00, 0.79]), which indicated full mediation, thus supporting H5 and H6. The effect of customer expressions on perceived severity was not significant [*M*_sadness_ = 2.93, SD = 1.49; *M*_anger_ = 2.83, SD = 1.43; *F*(1, 158) = 0.49, *p* = 0.485], and the indirect effect of perceived failure severity included zero (95% CI [−0.02, 0.10]), ruling out the confounding explanation.

**Table 1:** The mediating tests in Study 2.

Dependent variable	Effect type	Effect	SE	95% *CI*	
				*LLCI*	*ULCI*
Extra-role recovery	Direct effect	–0.25	0.18	−0.61	0.11
	Indirect effect of employee anger	0.19	0.08	0.05	0.39
	Indirect effect of employee other-concern	0.44	0.13	0.21	0.73
In-role recovery	Direct effect	−0.23	0.13	−0.50	0.04
	Indirect effect of employee anger	0.20	0.06	0.10	0.33
	Indirect effect of employee other-concern	0.12	0.05	0.04	0.22

To test H7, we compared the unstandardized coefficients of the path from other-concern emotions to in-role recovery (*b* = 0.12, SE = 0.05, *p* < 0.05) and extra-role recovery (*b* = 0.44, SE = 0.13, *p* < 0.01) following (Cohen et al., 2013). A *t*-test confirmed that the two coefficients differed significantly [*t*(159) = 2.36, *p* < 0.05], which showed that other-concern emotions exerted a stronger indirect effect on extra-role than on in-role recovery, thus supporting H7.

#### 3.2.5 Additional test

To assess the robustness of the findings across different respondent backgrounds, we conducted supplementary analyses examining the potential influence of gender and age on the observed effects (Dixon et al., 2017). Gender was examined using two-way ANOVA given its categorical nature, while age was examined using regression analysis. Results are summarized in Table 2. Results are summarized in Table 2.

**Table 2:** Respondent background analysis.

Test	Significance
Dependent variable = employees’ anger	
Gender	
A. Customer expression (anger/sadness)	*p* < 0.001.
B. Gender (female/male)	ns
A × B	ns
Age	
A. Customer expression (anger/sadness)	*p* < 0.001.
B. Age	ns
A × B	ns
Dependent variable = Employees’ other-concern	
Gender	
A. Customer expression (anger/sadness)	*p* < 0.001
B. Gender (female/male)	ns
A × B	ns
Age	
A. Customer expression (anger/sadness)	*p* < 0.001
B. Age	ns
A × B	ns
Dependent variable = extra-role recovery	
Gender	
A. Customer expression (anger/sadness)	*p* < 0.05
B. Gender (female/male)	*p* < 0.05
A × B	ns
Age	
A. Customer expression (anger/sadness)	*p* < 0.05
B. Age	ns
A × B	ns
Dependent variable = in-role recovery	
Gender	
A. Customer expression (anger/sadness)	ns
B. Gender (female/male)	*p* < 0.10
A × B	p < 0.05
Age	
A. Customer expression (anger/sadness)	ns
B. Age	ns
A × B	ns

ns represents non-significant difference at *p* > 0.10.

The pattern of results was consistent with the main analyses across both demographic variables. The main effect of customer emotional expressions on employees’ reactive emotions and extra-role recovery remained significant across gender and age groups, while the non-significant effect on in-role recovery was equally replicated, both patterns aligning with the hypothesized results. Neither gender nor age produced significant interaction effects with customer expression on employees’ reactive emotions, confirming the robustness of H1 and H2 across respondent backgrounds. Age showed no significant main effect or interaction with customer expression on any recovery outcome.

For recovery behaviors, gender showed a significant interaction with customer expression on in-role recovery (*p* < 0.05) and a significant main effect on extra-role recovery (*p* < 0.05). Exploratory follow-up analyses revealed that male employees demonstrated greater sensitivity to customer emotional expressions in their recovery behaviors. Specifically, when facing an angry customer, male employees reported lower in-role recovery (*M*_anger_ = 5.63, SD = 1.06; *M*_sadness_ = 6.05, SD = 0.71; *p* < 0.05) and extra-role recovery (*M*_anger_ = 3.86, SD = 1.49; *M*_sadness_ = 4.63, SD = 1.15; *p* < 0.05) compared to when dealing with a sad customer. Such a pattern was less evident among female employees. These finding suggest that while employees’ reactive emotions toward customers are robust across gender, female employees appear better able to maintain consistent recovery performance independent of the customer’s emotional expression. This gender difference is discussed further in the following section.

## 4 Discussion

This research examines how customer sadness and anger expressions influence employees’ emotional and behavioral responses in service failure and recovery contexts. Drawing on appraisal theory, our findings show that employees evaluate these two emotions distinctly in the primary appraisal stage, generating qualitatively different emotional reactions: higher other-concern emotions toward sad customers and relatively higher anger toward angry customers. Notably, the absolute level of anger remains lower than that of other-concern emotions across conditions, likely reflecting professional role expectations that condition employees to regulate negative affect and prioritize socially appropriate responses. In the secondary appraisal stage, these emotional reactions are further shaped by role-based coping constraints. That is, in-role recovery remains consistent across conditions as employees adhere to service norms, while extra-role recovery is strategically modulated, with employees responding more proactively to sad customers and more restrainedly to angry ones. Taken together, these findings underscore that employee do not simply mirror customer emotions in their behavior, but rather regulate and transform their affective responses, navigating the tension between emotional contagion and professional demands.

### 4.1 Theoretical implications

Our research provides several theoretical contributions to the study of service failure and recovery in customer-employee interactions. First, this research distinguishes between two types of negative customer emotional expressions, anger and sadness, and examines their differential interpersonal influences on employees within service failure and recovery encounters. Prior researchers have extensively explored the dynamic impacts of customer anger on service employees (e.g., Glikson et al., 2019; Tronvoll, 2011), but has largely overlooked the variation on emotion type (Van Kleef, 2016). Our work highlights customer sadness as a distinct emotional cue is different from anger, and can communicate vulnerability and support-seeking. By taking a comparative, fine-grained perspective, we show that employees can discern different, even opposite, social information from customer angry and sad expressions, for example the opposing attributions of blame versus support-seeking intent. Thus, we broaden the scope of existing literature and contributing to the pioneering attempts to explore how discrete negative customer emotions shape employees’ service outcomes within dyadic interactions.

Second, this research advances understanding of how and why different customer emotional displays impact employees’ recovery behaviors differentially. By explicitly distinguishing recovery type, we show that sad expression holds a clear advantage over angry expression in promoting autonomous softer extra-role recovery, but not must-do in-role recovery. Most existing studies focus on identifying effective recovery tactics that employee *should do* to retain dissatisfied customers (Gelbrich, 2010; Menon and Dubé, 2007), our research reveals what employees *would do*, showing that they strategically adjust their responses based on emotional reciprocity and the coping constraints imposed by their professional roles (Schepers and van der Borgh, 2020; Van Vaerenbergh et al., 2019). Our study thereby extends the application of two-stage appraisal theory and emotional reciprocity to service failure and recovery encounters (Marinova et al., 2018; Yue et al., 2020). Moreover, while prior studies have documented both negative (Rafaeli et al., 2012; Rupp and Spencer, 2006) and positive (Hareli et al., 2009; Miron-Spektor et al., 2011) effects of customer anger on employees, our differentiation between discretionary and role-prescribed recovery behaviors helps reconcile these seemingly contradictory findings. These insights contribute to a broader understanding of how emotional labor operates differentially across mandatory and discretionary service behaviors, a distinction that recent scholarship on emotional labor in service industries has identified as a key direction for future inquiry (Yoo et al., 2025). Additionally, our supplementary analysis revealed that gender moderates the relationship between customer emotional expressions and employees’ recovery behaviors, with male employees showing greater sensitivity to customer anger in both in-role and extra-role recovery. This finding is consistent with research on gender differences in emotional regulation and display rules (Goubet and Chrysikou, 2019; Nolen-Hoeksema, 2012), suggesting that female employees may be more adept at suppressing negative emotional reactions in professional contexts due to socialization processes that emphasize communal and care-oriented behaviors (Hochschild, 1983). This gender-based boundary condition adds nuance to our understanding of how professional role expectations interact with individual-level characteristics to shape service recovery outcomes.

Third, our research applies two-staged appraisal theory to elucidate the interpersonal impact of customer negative emotions on employees’ reactions, thus complementing prior research that has predominantly focused on the path from customers’ event appraisals to their own reactive emotions and coping strategies (e.g., Gelbrich, 2010; González-Gómez et al., 2021). Our work shifts attention to the reciprocal interaction between customer and employee emotions, showing that pre-existing customer emotions expressed as anger or sadness, can serve as the initial input for employees’ appraisal and coping processes, which in turn guide their remedial actions. This perspective is consistent with recent integrative frameworks that conceptualize emotion as simultaneously a cause, effect, mediator, and moderator in service contexts (Aeron and Rahman, 2025), and our study contributes an interpersonal-level analysis to this growing body of work.

Fourth, this research also contributes to literature on compassion in service contexts by examining how employees’ altruistic responses unfolds within interpersonal interactions (Dutton et al., 2014). Past research considers altruism as a static element that employees should internalize into regular work (Koerner, 2000). Adopting a process perspective, our study uncovers how suffering sad customers initiate employees’ other-concern affection, and how it is manifested in autonomous recovery behaviors. The findings bring a new process view to traditional research on employee altruism in the service field (Wirtz and Jerger, 2016; Chan and Wan, 2012), and resonate with recent evidence that customer interactions can serve as a source of emotional energy for frontline employees rather than merely depleting it (Cayla and Auriacombe, 2025).

Furthermore, this research underscores the essential role of human emotional engagement in service interactions, with implications for the growing literature on human versus technical frontlines in the era of artificial intelligence (Luo et al., 2025; Jose et al., 2026; Vorobeva et al., 2022). As AI-assisted systems take over routine service tasks, the capacity to distinguish between a sad and an angry customer and calibrate recovery effort accordingly remains a distinctively human competency. This highlights the irreplaceable role of human employees in emotionally complex encounters, and calls for future research on how AI systems can be designed to support, rather than supplant, empathic and adaptive recovery behaviors.

### 4.2 Practical implications

This study reveals that employees react differently to customer negative emotions. Employees’ emotional and recovery responses vary in a fine-grained manner depending on whether customers display anger or sadness. Recognizing that anger and sadness cue distinct response patterns enables organizations to design more targeted training and management interventions. By understanding these nuances, managers can better anticipate and address the challenges inherent in the service failure interaction. The findings yield two sets of practical implications, targeting anger versus sadness conditions, respectively.

Our findings show that customer anger specifically suppresses employees’ discretionary extra-role recovery behaviors through defensive reciprocity, while leaving mandatory in-role recovery largely intact. This asymmetry has direct implications for training design. Rather than broadly encouraging employees to do more for angry customers, managers should target the specific gap between in-role and extra-role performance under anger conditions. Two practical approaches are recommended. First, reframing exercises can help employees attribute customer anger to situational frustration rather than personal attack, thereby attenuating the defensive reciprocity response identified in this study (Luo et al., 2021). This can be implemented through structured team debriefs, one-on-one coaching sessions, or scenario-based training modules that draw on real service interactions. Second, scenario-based role-playing that simulates angry customer interactions can build employees’ capacity to sustain extra-role efforts even when feeling defensive. Explicit coaching should focus on maintaining discretionary helping behaviors (e.g., proactive problem-solving and personalized assistance) that our data show most at risk under anger conditions. Notably, our additional analysis found that male employees seem particularly susceptible to anger customers and thus reduce their recovery performance. Given this, the reframing and role-playing interventions outlined above may be especially valuable for male frontline staff.

Second, our results also demonstrate that customer sadness activates employees’ other-concern emotions, which in turn drive spontaneous extra-role recovery. Managers can leverage this mechanism in two ways. First, emotional recognition training that helps employees identify subtle cues of customer sadness, such as softened voice, downward gaze, and resigned language, can strengthen the other-concern activation process and its downstream effect on extra-role helping (Nijjer et al., 2026). Second, recognition and reward systems that make extra-role recovery behaviors visible and valued can reinforce the prosocial motivation that our data show is naturally present when employees encounter sad customers.

### 4.3 Limitations and future research

First, the experimental stimuli used in this research have inherent limitations in representing the complexity of real-world service interactions. In Study 1, customer emotions were manipulated solely through static facial images drawn from a standardized database (i.e., NimStim), and Study 2 supplemented these with a single verbal statement. However, emotional expressions in actual service encounters are inherently multimodal, encompassing facial cues, vocal tone, verbal content, and bodily posture simultaneously (Kranzbühler et al., 2020). The reliance on facial images alone may underrepresent this richness and potentially attenuate the intensity of emotional responses elicited from participants, particularly high-arousal emotions such as anger, which are typically accompanied by strong vocal and postural cues (Russell, 2003). More broadly, scenario-based experiments, while effective for maintaining experimental control and minimizing memory bias, may not fully replicate the dynamic and reciprocal nature of live service interactions. Future research could address these limitations by employing video-based stimuli, behavioral observation in field settings, or immersive virtual reality simulations to more authentically capture the multimodal character of customer emotional expressions and elicit more ecologically valid responses from employees.

Second, while our findings demonstrate regulated emotional reciprocity, where employees respond with a mix of anger and other-concern emotions rather than direct emotion mirroring, the relatively low reported anger levels suggest a potential influence of emotional display rules (Hochschild, 1983). Employees in customer service roles are typically expected to regulate negative emotions, which may have led to self-reported underestimation of anger. Future studies could employ physiological measures (e.g., heart rate variability, facial expression analysis) or observer-rated emotional assessments to obtain a more objective measurement of employees’ emotional states.

Third, we uncovered the process by which expressions of anger and sadness affect employees’ service outcomes; however, more research is needed to address the potential moderators, including individual-level factors (i.e., employees’ personality, disposition, and emotional intelligence), relation-level factors (i.e., similarity, closeness, and power difference), and organization-level factors (i.e., shared value, service climate, and leadership style). Future research should also examine employees’ professional experience and tenure as individual-level moderators, as seasoned employees may respond to customer anger and sadness differently from novices due to differences in emotional display rules and accumulated coping repertoires.

Lastly, although employees’ other-concern emotions have the advantages on their altruistic responding, but practitioners must consider the potential cost. For example, altruistic employees may become over-concerned with customers’ well-being, even at the cost of organizational benefits (Yagil and Medler-liraz, 2013). Customers may also find a certain degree of over-involvement inappropriate and respond with social rejection (Marinova et al., 2018). Future research can investigate the possible dark side of employees’ other-concern.

## Data Availability

The original contributions presented in the study are included in the article/Supplementary material, further inquiries can be directed to the corresponding author.
