# Supplementary material for: How employees respond to customer anger and sadness: emotional reciprocity and recovery strategies in service failures
Source: Front Psychol. 2026 May 20;17:1795506. doi: 10.3389/fpsyg.2026.1795506 (PMC13229742; doi:10.3389/fpsyg.2026.1795506)
Supplement: Supplementary file 1 [file Supplementary_file_1.docx]

**Appendix A. Scenarios of Study 1 and Study 2**

| Study 1 (adapted from Gelbrich (2010)) | |
| --- | --- |
| Imagine you are a hotel receptionist. You are behind the desk and a customer is walking towards you. He tells you that he is on a 3-day sightseeing trip with his girlfriend for the summer holiday and comes to complain because, on the first day in the hotel, a constant loud noise woke them up at 5 a.m. in the morning.  *(The story continues on the next page, where you will also see the customer. Please watch his reactions and read carefully.)* | |
| Study 2 (created for this research) | |
| Imagine you are a hotel receptionist, at a 3-star tourist hotel. You are behind the counter and a customer is walking towards you. He tells you that he has spent three nights in the hotel. The price includes access to the hotel's fitness center and pool, which he was very much forward to use.  *(The story continues on the next page, where you will also see the customer. Please watch his reactions and read carefully.)* | |

**Appendix B. Manipulations of customer expression in Study 1 and Study 2**

| Anger Expression | Sadness Expression | Neutrality Expression |
| --- | --- | --- |
| 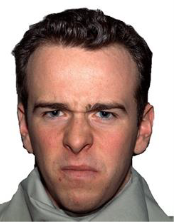 | 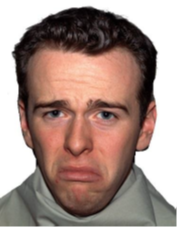 | 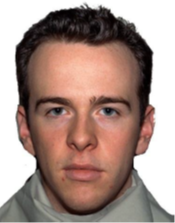 |

Note: Study 1 comprised three conditions with three pictures. Study 2 designed two conditions using the pictures of anger expression and sadness expression, with the exception of neutrality expression.

**Appendix C. Measurement Items and Reliability in Study 1 and Study 2**

| **Measures (Scale Sources; Reliability)** | | |
| --- | --- | --- |
| **In-role recovery behaviors** (van der Heijden et al. (2013); Study 2: Cronbach α = 0.82) | | |
| *Facing the consumer before me, I would …* | |  |
| 1. make an effort to ensure that the consumer could sleep undisturbed tonight (use the pool today, in Study 2). | |  |
| 2. try my best to efficiently solve the customer’s problem. | |  |
| 3. treat him considerately and respectfully. | |  |
| 4. serve him in a courteous manner. | |  |
| 5. be polite to him. | |  |
| **Extra-role recovery behaviors** (Chan and Wan (2012); Study 2: Cronbach α = 0.90) | | |
| *Facing the customer before me, I would …* | |  |
| 1. go out of my way to anticipate and satisfy his needs. | |  |
| 2. go above and beyond the “call of duty” when serving him. | |  |
| 3. voluntarily assist him even if it means going beyond job requirements. | |  |
| 4. help him beyond what is expected or required in our task. | |  |
| **Employees’ anger emotion** (Du et al. (2014); Study 1/2: Cronbach α = 0.89/ 0.89) | | |
| *Given the way the consumer interacted with me, to what extent would you …* | |  |
| 1. feel angry toward him. | |  |
| 2. feel displeased toward him. | |  |
| 3. feel irritated toward him. | |  |
| 4. feel furious toward him. | |  |
| **Employees’ other-concern emotions** (van Kleef et al. (2008), Sinaceur et al. (2015); Study 1/2: Cronbach α = 0.89/ 0.95) | |  |
| *Given the way the consumer interacted with me, to what extent would you …* | |  |
| 1. feel compassion for him. | |  |
| 2. feel empathy toward him. | |  |
| 3. feel sympathy toward him. | |  |
| **Perceived Severity of Scenario** (Grégoire et al. (2009); Study 2: Cronbach α = 0.91)  1. The service failure is: a minor problem – a major problem  2. The service failure caused is: small convenience – big convenience | | |
| 3. The service failure caused is: a minor aggravation – a major aggravation | | |
| **Manipulation check** (Cheshin et al. 2018) | | |
| To what extent did the customer seem … | |  |
| 1. angry | |  |
| 2. sad | |  |
| How intense was the customer’s emotional expressions? | |  |
